# Duplicated Appendix With Appendicitis and Appendicolith: A Case Report of a Rare Clinical Encounter

**DOI:** 10.7759/cureus.89619

**Published:** 2025-08-08

**Authors:** Ashley Cardona, Karla Oliveira, Ioana Andreea Rezac, Logan J Jenkins, Imtiaz Ahmed

**Affiliations:** 1 Radiology, Universidad Autónoma de Guadalajara School of Medicine, Guadalajara, MEX; 2 Radiology, Avalon University School of Medicine, Willemstad, CUW; 3 Radiology, Midwestern University Arizona College of Osteopathic Medicine, Glendale, USA; 4 Radiology, Tempe St. Luke's Hospital, Tempe, USA

**Keywords:** acute appendicitis, appendicolith, appendix, cave-wallbridge classification, computed tomography, congenital malformation, duplicated appendix

## Abstract

Duplication of the appendix is a rare congenital malformation, classified into various types depending on its location and relation to the cecum. There are no established demographic patterns associated with appendiceal duplication. While the exact cause and mechanism are not entirely understood, duplication of the appendix is thought to arise between weeks six and eight of gestation due to anomalies during the embryological process of midgut rotation. We present an unusual case of a 26-year-old male patient who presented to the ED with a 12-hour history of nausea, vomiting, and right lower quadrant (RLQ) abdominal pain. The physical examination revealed McBurney’s point tenderness, guarding, and rigidity. Laboratory findings showed leukocytosis with a WBC of 14,000/μL (reference range: 4,000-11,000/μL). Following a contrast-enhanced computed tomography (CT), a diagnosis of a duplicated appendix, one of them with acute appendicitis and appendicolith, was established. This case highlights the importance of considering appendiceal duplication in patients with appendicitis-like symptoms and the utility of CT in diagnosis.

## Introduction

The appendix (vermiform appendix) is a narrow, blind-ended tubular structure that originates from the posteromedial wall of the cecum, near the confluence of the taeniae coli [1,2]. It has an average length of 9 cm and receives its blood supply from the appendicular artery, a branch of the ileocolic artery [1]. While historically considered a vestigial organ, it has been proposed to play a role in gut-associated lymphoid tissue (GALT), contributing to immune function and serving as a potential reservoir for gut microbiota [3]. The appendix is intraperitoneal, with variable positions including retrocecal, pelvic, subcecal, preileal, and postileal [2,4]. Duplication of this organ is an exceptionally rare congenital anomaly, with literature reporting an incidence of one in 25,000 [5,6]. It is categorized using the Cave-Wallbridge classification system into four types: A, B1, B2, and C [7]. Such cases can present a significant clinical challenge, particularly during routine appendectomies, where an unrecognized duplicated structure may lead to incomplete treatment or future diagnostic challenges. Duplicated appendices, particularly those in types B1 and C, are often associated with other congenital malformations [5]. Diagnosis typically occurs once symptoms are present or as an incidental finding during imaging for unrelated conditions. There is no clear dominance regarding gender, race, age, or body mass index (BMI) in the occurrence of this anomaly [6].

We present a rare case of appendiceal duplication, complicated by acute appendicitis and an appendicolith in one of the appendices, emphasizing the diagnostic challenges and intraoperative considerations associated with this anomaly. This case underscores the importance of computed tomography (CT) for accurately detecting appendiceal malformations and highlights the need for heightened surgical awareness to optimize patient outcomes.

## Case presentation

A 26-year-old male presented to the emergency department with a 12-hour history of progressively worsening right lower quadrant (RLQ) abdominal pain, accompanied by loss of appetite, nausea, and vomiting. The patient described an acute onset of abdominal pain, initially localized to the periumbilical region, which subsequently migrated to the RLQ, where it intensified and assumed a sharp quality. The pain was exacerbated by movement, including coughing, sneezing, and ambulation. Prior to presentation, he had taken 400 mg of ibuprofen at home, which failed to provide symptomatic relief. He endorsed a generalized sense of malaise and intermittent chills, with no fever. The patient reported no history of chronic medical conditions, no use of alcohol, tobacco, or recreational drugs, and no relevant family history of gastrointestinal or abdominal pathology. There were no recent changes in diet, travel history, or exposure to infectious contacts. On further review, he recalled experiencing mild RLQ discomfort the previous day, which had resolved spontaneously and did not prompt medical evaluation.

**Figure 1:**
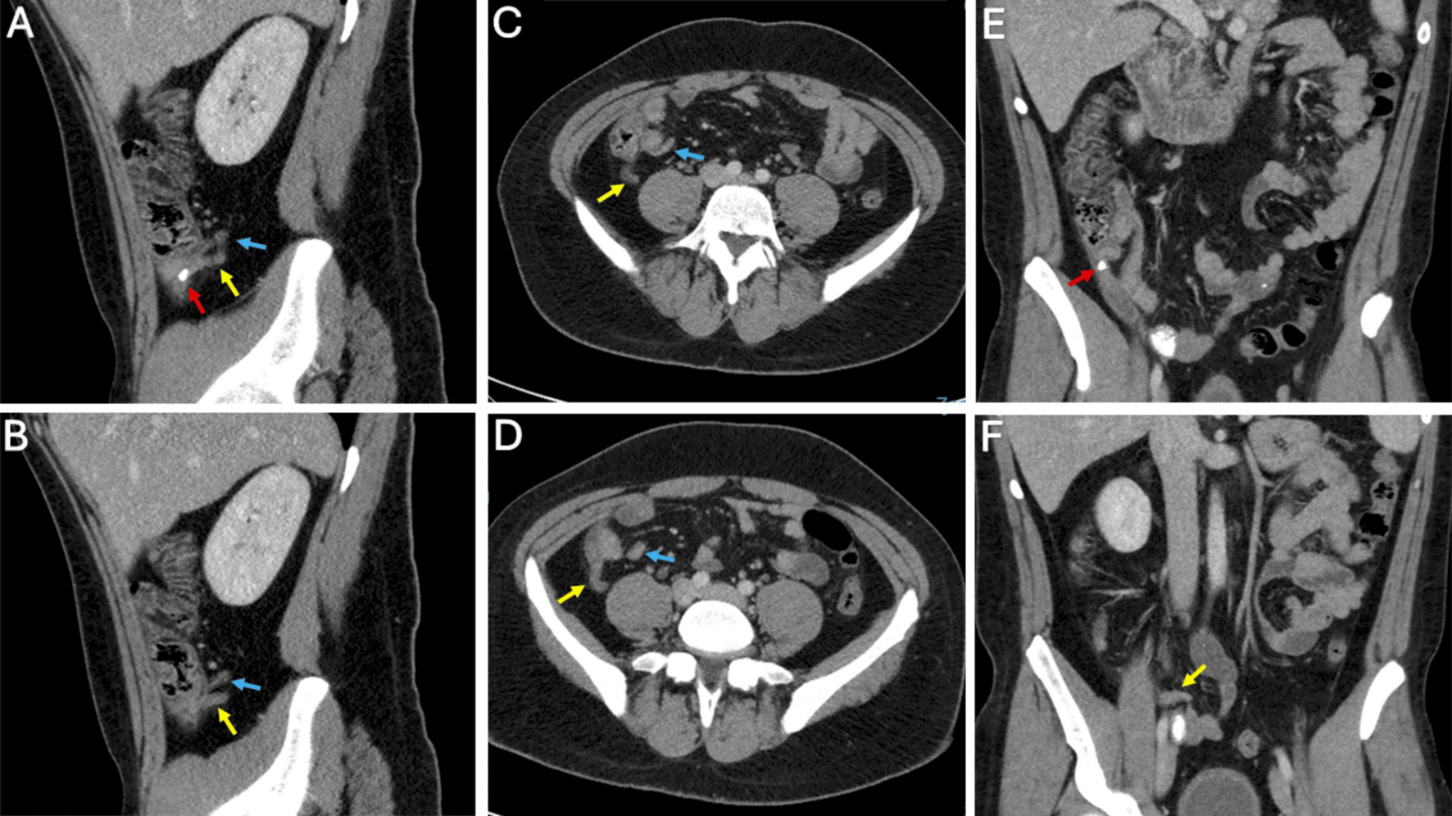
Contrast-enhanced computed tomography scan revealing a thickened and dilated appendix (yellow arrow) with appendicolith (red arrow), as well as a second appendix (blue arrow) in a type B2 configuration. Sagittal (A, B), axial (C, D), and coronal (E, F) views are shown.

Vital signs were as follows: blood pressure of 138/88 mmHg, heart rate of 104 bpm, and temperature of 38°C. Physical examination revealed McBurney’s point tenderness, accompanied by guarding and rebound tenderness. Laboratory findings showed leukocytosis with a WBC of 14,000/μL (reference range: 4,000-11,000/μL). A contrast-enhanced CT scan was performed, which revealed two separate appendices: one showing signs of acute inflammation and an appendicolith within its lumen. The second appendix appeared normal, with no signs of inflammation, as shown in Figure 1. A diagnosis of a type B2 duplicated appendix, with one appendix complicated by acute appendicitis and appendicolith, was established. The patient underwent a laparoscopic appendectomy. During surgery, the preoperative diagnosis was confirmed, and both appendices were excised. Histopathological evaluation corroborated these findings, demonstrating acute suppurative inflammation characterized by transmural neutrophilic infiltration and mucosal ulceration in the affected appendix, confirming the diagnosis of acute appendicitis, while the second appendix showed no abnormalities. The patient recovered without complications and was discharged on the third postoperative day.

## Discussion

Duplication of the appendix, first described by Piccoli in 1892, is a rare congenital malformation, with just over 100 cases reported in the medical literature. According to estimates, the incidence of this anomaly ranges from 0.004% to 0.009% [6]. The Cave-Wallbridge classification system, currently the most widely accepted, categorizes appendiceal duplications into four types: A, B1, B2, and C [5]. These types are distinguished based on the location and relationship of the duplicated appendices to the cecum. Type A has a single cecum and one appendix exhibiting various degrees of partial duplication. Type B1 has two appendices symmetrically placed on either side of the ileocecal valve. Type B2 has one appendix that arises from the cecum at the usual site, and the second branches at varying distances along the lines of the taenia from the first. Type C has a double cecum, each bearing its own appendix [7]. The type of duplication that develops is contingent on specific disruptions in the normal embryological process of midgut rotation during weeks six to eight of gestation [2,5,8]. Type A results from the fusion of the appendix precursor with the temporary appendix-like structure. Type B1 is caused by a failure in cloacal differentiation, while type B2, the most prevalent, stems from the proliferation of the temporary appendix-like structure, which normally regresses by week eight. Type C occurs due to inadequate differentiation of the cloaca [5,8].

Types A and B2 typically remain undetected. Meanwhile, types B1 and C are associated with additional congenital anomalies, such as hindgut atresia, hindgut duplication, ectopic bladder, enterovesical fistula, duplication of reproductive organs, and imperforate anus [5,6]. As a result, types B1 and C are likely to be discovered incidentally during surgical management for concomitant conditions.

The presence of a second appendix may increase the likelihood of developing inflammation or obstruction [9,10]. It may also predispose to appendicolith formation, which increases the risk of localized obstruction, infection, and perforation [11]. CT is one of the primary diagnostic modalities given its high sensitivity and specificity. Typical findings on CT to diagnose acute appendicitis include evidence of an enlarged appendix, usually exceeding 6 mm in diameter, accompanied by thickening of the appendiceal wall and signs of inflammation such as fat stranding or free fluid around the appendix [12]. Ultrasound is often preferred in patients where ionizing radiation exposure should be limited, such as pediatric or pregnant patients [6]. As a limitation, ultrasound can fail to recognize a second appendix, particularly in the setting of a retrocecal appendix [6,9].

Appendicitis is the most common cause of emergency surgeries. If left untreated, it can cause abscess formation, acute abdomen, sepsis, and death [1]. In the setting of acute appendicitis in a patient with a duplex appendix, treatment generally follows the same approach as in patients with normal anatomy. An appendectomy is the treatment of choice, with some authors advocating for the excision of both appendices to prevent future complications, such as recurrent appendicitis, diagnostic confusion, or misinterpretation of imaging studies [5,9]. Consistent with these considerations, both appendices were removed from our patient. Histopathological evaluation confirmed acute suppurative appendicitis in one appendix, while the second showed no pathological abnormalities, supporting our diagnosis. It is important to note that, in patients with a history of prior appendectomy, a second appendix may have been overlooked, leading to diagnostic confusion. The residual appendix may remain unnoticed during imaging or surgery, potentially delaying diagnosis and treatment. Awareness of this anomaly is therefore essential for clinicians when evaluating patients presenting with RLQ pain, even if an appendectomy has been previously performed. To contextualize our case within the published literature, a comparison with previously reported cases of appendiceal duplication complicated by acute appendicitis is provided in Table 1 to highlight both the similarities and distinguishing features of this case.

**Table 1:** Comparison of published cases of appendiceal duplication complicated by acute appendicitis. Abbreviations: Computed tomography (CT), Ultrasound (US), Postoperative day (POD) ¹ Initial emergency presentation; ² Second emergency presentation, after two months, for unrelated symptoms (constipation). Data derived from Tinkham et al. [6], Ayoub et al. [9], and Aslaud et al. [10].

Author/Year	Present Case	Tinkham et al. [6], 2021	Ayoub et al. [9], 2018	Aslaud et al. [10], 2023
Patient Age / Biological Sex	26 / M	10 / M	30 / F	36 / M
Type	B2	B2	B1	B2
Diagnosis Method and Findings	CT: both appendices identified; one inflamed with appendicolith	US: one inflamed appendix identified¹; CT: incidental second appendix with no signs of inflammation²	US: appendiceal shield	US: one inflamed appendix identified; duplication found intraoperatively
Surgical Approach	Laparoscopy: both removed	Laparoscopy: one appendix with appendicolith removed¹; no surgery²	Laparotomy: both removed	Laparotomy: both removed
Histopathology	First appendix inflamed with appendicolith; second with no abnormalities	Not available¹²	First appendix inflamed; second with no abnormalities	Both appendices inflamed, each containing an appendicolith
Outcome	Uneventful recovery; discharged POD 3	Uneventful recovery¹; appendix noted on CT²	Uneventful recovery; discharged POD 3	Uneventful recovery

## Conclusions

This case highlights a rare presentation of type B2 appendiceal duplication, complicated by acute appendicitis with appendicolith in one of the appendices. Such anomalies can present significant diagnostic and surgical challenges, particularly if unrecognized. Preoperative imaging, especially contrast-enhanced CT, is instrumental in identifying anatomical variants and guiding appropriate surgical intervention. Complete removal of both appendices is recommended to avoid future complications, including recurrent appendicitis and diagnostic confusion. Increased awareness of this congenital anomaly among clinicians may contribute to improved diagnostic accuracy and optimized patient outcomes.

## References

[1] Hodge BD, Kashyap S, Khorasani-Zadeh A (2023). Anatomy, abdomen and pelvis: appendix. StatPearls.

[2] Schumpelick V, Dreuw B, Ophoff K, Prescher A (2000). Appendix and cecum: embryology, anatomy, and surgical applications. Surg Clin North Am.

[3] Kooij IA, Sahami S, Meijer SL, Buskens CJ, Te Velde AA (2016). The immunology of the vermiform appendix: a review of the literature. Clin Exp Immunol.

[4] Ghorbani A, Forouzesh M, Kazemifar AM (2014). Variation in anatomical position of vermiform appendix among Iranian population: an old issue which has not lost its importance. Anat Res Int.

[5] Nageswaran H, Khan U, Hill F, Maw A (2018). Appendiceal duplication: a comprehensive review of published cases and clinical recommendations. World J Surg.

[6] Tinkham M, Ziesat M, Straumanis J, Heisler S (2021). Double appendix: implications for the emergency department. J Emerg Med.

[7] Wallbridge PH (1962). Double appendix. Br J Surg.

[8] Bluett MK, Halter SA, Salhany KE, O'Leary JP (1987). Duplication of the appendix mimicking adenocarcinoma of the colon. Arch Surg.

[9] Ayoub K, Kayali S, Dabbagh MF, Banjah B (2018). Acute single appendicitis in a female with a duplicated appendix. J Surg Case Rep.

[10] Alsaud JS, Alnumayr H, Aljamaan S, Aloufi M, Momtaz A (2023). Acute appendicitis in a double appendix: a case report. Cureus.

[11] Kaewlai R, Wongveerasin P, Lekanamongkol W (2024). CT of appendicoliths in adult appendicitis: clinical significance and characteristics of overlooked cases. Eur Radiol.

[12] Wonski S, Ranzenberger LR, Carter KR (2023). Appendix imaging. StatPearls.

